# New Year's silent killer in Japan: Undigested mochi

**DOI:** 10.1002/ccr3.6682

**Published:** 2022-12-05

**Authors:** Koki Kawanishi, Yoshifumi Ikeda

**Affiliations:** ^1^ Department of Gastroenterology Nate Hospital Kinokawa Japan

**Keywords:** endoscopic procedure, endoscopy, foreign substance, mochi, rice cake

## Abstract

Swallowed rice cake (mochi) without chewing retained in the stomach intactly. We successfully treated the hardened mochi without surgery. Eye‐catching images showed as many as 10 intact mochi in the stomach and endoscopic crushing procedure.

## Abstract 2

Misconception that gastric acid can dissolve rice cake (mochi) leads to retention of mochi in the stomach. Although mochi is soft in a hot soup dish, it can get much harder in the stomach temperature. Swallowed mochi without chewing can be resolved only by endoscopic procedure.

## 1 CLINICAL IMAGE

**FIGURE 1:**
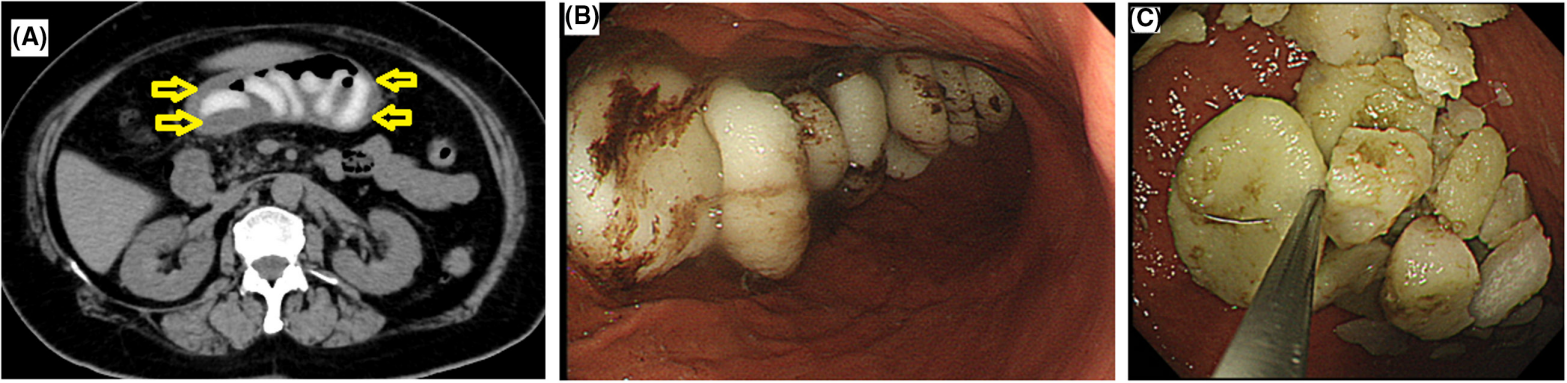
(A) CT revealed multilayer arc‐like high‐density objects in the stomach. (B) Endoscopy revealed 10 completely intact round mochi. (C) We endoscopically sliced the mochi to <10 mm by snaring without electrical current.

A 65‐year‐old woman presented to our hospital with a complaint of abdominal pain. Abdominal computed tomography (CT) revealed multilayer arc‐like high‐density objects in the stomach (Figure 1A, yellow arrows). The patient had eaten several pieces of 30‐mm rice cake (mochi) in a Japanese traditional soup dish without enough chewing. Mochi is made of short‐grain japonica glutinous rice that becomes soft and extremely sticky after boiling or toasting. It is an indispensable food in Japan, especially in the New Year's holiday but has caused numerous deaths by suffocation among elderly people every year because of its stickiness.^1^ In the present case, emergency endoscopy revealed 10 completely intact round mochi (Figure 1B), which remained intact after as long as 5 days. The stomach temperature stiffens mochi to an extent indissoluble by gastric juice. We endoscopically sliced the mochi to <10 mm by snaring without electrical current (Figure 1C). Digestive enzyme (Excelase 3.0 g/day, Meiji Seika Pharma Holdings Co, Ltd) was prescribed. Two days after snare cutting, endoscopy revealed that all the mochi had disappeared, and abdominal CT revealed no residual mochi in the intestine. Ten‐millimeter mochi can be drained to the duodenum from the pylorus ring, where it can then be dissolved by bile and pancreatic juice.^2^

Although airway obstruction by mochi is well recognized, a gastro‐intestinal foreign body associated with mochi is poorly recognized because of its rarity. Retained mochi can cause severe gastric ulcer which possibly leads to gastric perforation. Accordingly, retained mochi requires an endoscopic procedure as soon as possible.^3^ Therefore, primary care physicians and emergency doctors, as well as gastroenterologists, should become familiar with the therapeutic methods for this rare but popular‐food‐related foreign body.

## AUTHOR CONTRIBUTION

All authors contributed significantly toward the completion of this case report.

## CONFLICT OF INTEREST

The authors declare no conflict of interest.

## ETHICAL APPROVAL

None.

## CONSENT

Written informed consent was obtained from the patient to publish this report in accordance with the journal's patient consent policy.

## Data Availability

The data that support the findings of this study are available on request from the corresponding author. The data are not publicly available due to privacy or ethical restrictions.
